# COVID-19 Vaccination a Cause of Guillain-Barré Syndrome? A Case Series

**DOI:** 10.7759/cureus.30888

**Published:** 2022-10-30

**Authors:** Govind Nagdev, Gajanan Chavan, Gaurav Sahu, Poosapati D Devasilpa Raju

**Affiliations:** 1 Department of Emergency Medicine, Jawaharlal Nehru Medical College, Datta Meghe Institute of Medical Sciences, Wardha, IND; 2 Department of Medicine, Jawaharlal Nehru Medical College, Datta Meghe Institute of Medical Sciences, Wardha, IND; 3 Department of Surgery, Jawaharlal Nehru Medical College, Datta Meghe Institute of Medical Sciences, Wardha, IND

**Keywords:** sars-cov-2 vaccination associated with gbs, molecular mimicry in covid-19, critical illness polyneuropathy, guillain-barre syndrome (gbs), covid-19 vaccine

## Abstract

Guillain-Barré syndrome (GBS) is a rare autoimmune neuropathic disorder of peripheral nerves usually following an infection or on rarer occasions following vaccinations, but the exact underlying pathophysiology is still unclear. The most common etiology of GBS is a bacterial infection caused by *Campylobacter jejuni*. Viral infections like Zika virus, Epstein-Barr virus, and Cytomegalovirus also add to the list of GBS etiology. COVID-19 (SARS-CoV-2) has also been reported to cause GBS. Vaccines like the rabies vaccine, influenza vaccine, and poliovirus vaccine account for a very small fraction of Guillain-Barré syndrome. GBS as an adverse effect of COVID-19 vaccination was not reported by the Vaccine Adverse Event Reporting System (VAERS), but an update was later released in the course of the pandemic from FDA news, reporting several patients developing GBS after receiving the COVID-19 vaccine. In this case series, we discuss five cases that developed the GBS post-COVID-19 AstraZeneca vaccine, along with its pathophysiology, management, and outcome.

## Introduction

Guillain-Barré syndrome (GBS) is a rare autoimmune polyradiculoneuropathy disorder of peripheral nerves presenting as progressive ascending paralysis with symmetrical limb weakness, hyporeflexia or areflexia, autonomic dysfunction, and sensory abnormalities. The global prevalence of GBS ranges from 0.6 to 4.0/per 100,000 person-years with an average mortality rate of 5% [1-3]. GBS is usually preceded by an infectious trigger by bacterial or viral agents most commonly associated with infection caused by *Campylobacter jejuni* (*C. jejuni*) [4]. In addition, recent infections by the Zika virus, Epstein-Barr virus (EBV), and Cytomegalovirus (CMV) have also been reported as antecedent causes of GBS [5-7]. Severe acute respiratory syndrome (SARS‐CoV‐2) infection, resulting in the coronavirus disease 2019 (COVID‐19) pandemic, is associated with Guillain‐Barré syndrome (GBS) [8-10]. Vaccine Adverse Event Reporting System (VAERS) defined vaccine-associated GBS as those with the onset of symptoms within six weeks after receiving the vaccine [11,12]. The first association between GBS and vaccination was brought to attention in 1976 following the Influenza vaccine [13]. SARS-CoV-2 vaccination-related adverse effects range from mild-moderate to severe neurological dysfunction. Though rare, these neurological side effects are increasingly recognized and reported. A systemic review discusses 18 patients with post-vaccination GBS that were reported in nine articles [14]. Limitations in articles were that Brighton criteria were not used to diagnose GBS which requires nerve conduction studies (NCSs) and CSF investigations. Brighton criteria, a diagnostic and risk assessment tool for GBS comprises four clinical attributes and three investigatory findings. NCS and CSF findings make up those three panels of investigation in the Brighton criteria. In our study, all five patients of GBS were diagnosed clinically and supported with CSF studies and NCSs.

## Case presentation

We discuss five cases that were reported to the Emergency Medicine Department (EMD) of Acharya Vinoba Bhave Rural Hospital (AVBRH), a tertiary care rural hospital situated in central India majorly catering to the population of the Vidarbha region of Maharashtra, India.

All five patients received a non-replicating viral-vector Oxford/AstraZeneca ChAdOx1 nCoV-19 (AZD1222) COVID-19 vaccine.

Case 1

A 40-year-old male presented to the EMD with a history of bilateral lower limb weakness for six days which was progressive. He had no associated symptoms like rash, fever, headache, or blurring of vision. No previous diarrheal illness or respiratory tract infection. About 12 days before these symptoms, he received the first dose of the SARS-CoV-2 vaccine. On general examination, the patient was afebrile, and vitals were: Heart rate (HR): 100/min, Blood pressure (BP): 110/70. Central nervous system (CNS) examination revealed no facial asymmetry and intact cranial nerves. Motor examination of lower limbs revealed loss of ankle and knee reflexes bilaterally with hypotonia, power grade 2/5 in both lower limbs. MRI brain and whole spine did not reveal any significant abnormality. CSF analysis showed high albumin (2074) with normal WBCs count. The nerve conduction study (NCS) was suggestive of demyelinating sensory-motor polyneuropathy. He was started with Intravenous immunoglobulins (IVIg), analgesics, and other supportive measures. There was a gradual improvement in his condition with an improvement in muscle power on all four limbs. His respiratory function remained stable throughout his hospital stay. 

Case 2

A 23-year-old female presented with complaints of bilateral lower limb weakness, tingling, and numbness in all four limbs for 15 days. He had no history of infection and received COVID-19 vaccination a month ago. General examinations were within normal limits. CNS examination revealed no facial asymmetry and normal cognitive function, normal cranial nerve examination, normal tone, power grade 3/5 in both lower limbs and 4/5 in upper limbs. All peripheral reflexes were absent, with paraesthesia in both lower limbs. CSF study revealed albuminocytological dissociation. NCS suggestive of demyelinating sensory-motor polyneuropathy involving both upper and lower limbs, along with distal segments being grossly affected. She showed tremendous improvement with IVIg with the attainment of 5/5 power on all four limbs. 

Case 3

A 20-year-old male presented to EMD with quadriplegia for eight days. There was a history of COVID-19 vaccination two weeks ago. General examinations were within normal limits. CNS examination showed grade 3/5 power in all four limbs with areflexia and flaccid tone. MRI showed minimal annular bulges of C3-4 and C4-5 discs without significant neural compression. The cervicodorsal cord was normal without any abnormal signal or cord compression. Screening of the dorsolumbar spine was unremarkable. CSF study showed elevated albumin and normal glucose level. NCS was suggestive of sensory-motor axonal polyradiculoneuropathy. He was diagnosed with GBS, and his condition eventually improved with IVIg and corticosteroid administration.

Case 4

A 25-year-old male presented with quadriparesis and tingling numbness in all limbs for eight days, associated with difficulty in breathing. There was no history of infection. He took the first dose of COVID-19 vaccination 15 days back. General examinations were within normal limits. CNS examination revealed normal higher functions, a power grade of 3/5 on bilateral lower limbs, and 3/5 on upper limbs with hyporeflexia. CSF study showed elevated protein and normal glucose levels. NCS showed a significant reduction in compound muscle action potential (CMAP) amplitude in all four nerves in the test (Median, Ulnar, Tibial, and Peroneal) and Sensory Never Action Potential (SNAP) amplitude in the Median and Ulnar nerves suggestive of sensory-motor axonal polyradiculoneuropathy. The patient's forced vital capacity (FVC) and negative inspiratory force (NIF) were below 15ml/kg and 10cm H_2_O, respectively, indicating the involvement of respiratory muscles, for which his airway was secured, and started on IV antibiotics and IVIG with supportive treatment. He responded only partially to IVIGs. His condition deteriorated, and was later put on a mechanical ventilator on volume control mode but eventually landed into sudden cardiac arrest despite all cardiopulmonary resuscitation measures patient could not be revived and was declared dead with the cause of death stated as GBS.

Case 5 

A 62-year-old male presented to EMD, with weakness in all four limbs, and an inability to sit and stand, which is associated with backache for 20 days. He received his first dose of vaccination a month ago. He was conscious and oriented, and his vitals were within normal limits. Grade 0 power in bilateral lower limbs, 2/5 in bilateral upper limbs. CSF examination showed elevated glucose level (164 mg/dl), elevated albumin levels (244 mg/dl), and total leucocyte counts (TLC) 0. NCS showed sensory motor axonal polyradiculoneuropathy. He was treated with IVIG, antacids, antibiotics, and physiotherapy. He responded well to the treatment and was discharged. 

For each patient in Tables 1, 2, we have listed the CSF findings and nerve conduction study findings, respectively, to strengthen the diagnosis of the GBS along with history and physical examination.

**Table 1:** CSF findings of the five cases that presented with sensory-motor weakness post-COVID-19 AstraZeneca Vaccine CSF: Cerebrospinal Fluid, RBC: Red Blood Cell, WBC: White Blood Cell, DLC: Differential Leucocyte Count Cerebrospinal fluid normal ranges: WBC: 0 – 5 cells/μl, CSF total protein: 15 to 60 mg/100 mL, CSF glucose: 50 to 80 mg/100 mL

CSF FINDING SUMMARY									
	Age/Sex	Opening pressure (cm of H_2_0)	Appearance	Protein (mg/dl)	Glucose (mg/dl)	Gram Stain	RBC Count	WBC count	Other
Case 1	40/M	Normal	Clear, colourless, transparent	2074	55	Negative	Occasional	32	DLC= 60% lymphocytes
Case 2	23/F	Normal	Clear, blood tinged, transparent	124	72	Negative	Plenty	03	DLC= Too low to comment on
Case 3	20/M	Normal	Clear, blood tinged, transparent	79	62	Negative	Plenty	03	DLC= Too low to comment on
Case 4	25/M	Normal	Clear, colourless transparent fluid	99	58	Negative	Occasional	11	DLC= Predominantly mononuclear cells seen
Case 5	62/M	Normal	Clear, colourless transparent fluid	244.1	164.6	Negative	16	00	-

**Table 2:** Nerve Conduction Study (NCS) reports of the cases 1-5 MNCS: Motor Nerve Conduction Study, SNCS: Sensory Nerve Conduction Study, DML: Distal Motor Latency, CMAP AMPLITUDE: Compound Muscle Action Potential, MCV: Motor Conduction Velocity, SDL: Sensory Distal Latency, SNAP: Sensory Nerve Action Potential, SCV: Sensory Conduction Velocity

Nerve Conduction Study										
		Median Nerve		Ulnar Nerve		Tibial Nerve		Peroneal Nerve		Inference
		R	L	R	L	R	L	R	L	
Case 1	DML (ms)	3.6	4.1	3.2	3.4	6.6	6.2	7.1	7.6	1. B/L Tibial and Peroneal Nerve DML Prolonged 2. B/L Tibial and Peroneal Nerve MCV Reduced
	CMAP AMPLITUDE (mV)	9.43	10.2	7.34	9.23	4.82	5.3	2.48	2.8	
	MCV(m/s)	51.0	63.2	53.2	54.2	33.2	36.4	30.6	31.3	
	SDL (ms)	2.1	1.9	2.2	2.1	-		-		-
	SNAP (µV)	51.3	53.2	17.6	16.9					
	SCV (m/S)	57.3	56.9	58.2	58.6					
	F-wave latency	F-min latency absent								-
Case 2	DML (ms)	6.2	5.3	4.8	5.8	7.3	6.9	8.2	7.9	1. B/L Median and Ulnar Nerve DML Prolonged 2. B/L Median and Ulnar Nerve CMAP Amplitude Reduced 3. B/L Tibial and Peroneal Nerve DML Prolonged 4. B/L Tibial and Peroneal Nerve CMAP Amplitude Reduced
	CMAP AMPLITUDE (mV)	3.2	2.5	3.1	2.8	3.4	2.6	1.2	1.3	
	MCV(m/s)	54.0	52.3	56.4	54.7	46.2	43.6	44.9	47.7	
	SDL (ms)	2.2	1.8	1.9	1.6	-		-		1. B/L Median and Ulnar Nerves SNAP amplitude Reduced
	SNAP (µV)	36.0	34.3	7.8	6.3					
	SCV (m/S)	59.4	61.2	56.4	59.2					
	F-wave latency	1. Prolonged in B/L Median and Ulnar Nerves. 2. Absent in B/L Peroneal & Tibial Nerves.								
Case 3	DML (ms)	4.2	4.1	3.3	2.9	5.3	6.0	6.2	5.4	1.B/L Peroneal and Tibial CMAP Amplitude Reduced
	CMAP AMPLITUDE (mV)	5.1	4.6	7.8	7.2	2.5	2.7	0.8	1.2	
	MCV(m/s)	54.3	55.6	57.8	56.3	46.2	49.4	43.5	48.1	
	SDL (ms)	1.4	1.9	2.1	2.2	-		-		1. B/L Median and Ulnar Nerves SNAP Amplitude Reduced 2. B/L Sural Nerves SNAP Amplitude Reduced
	SNAP (µV)	42.4	40.1	5.8	7.6					
	SCV (m/S)	56.4	58.2	58.1	56.5					
	F-wave latency	1. Prolonged in B/L Median and Ulnar Nerves 2. Absent in B/L Peroneal & Tibial Nerves.								
Case 4	DML (ms)	5.6	5.5	4.8	4.9	6	5.9	6.1	6	1. B/L Median and Ulnar Nerve CMAPs Amplitude Reduced 2. B/L Median and Ulnar Nerve DML Prolonged 3. B/L Tibial and Peroneal Nerve CMAP Amplitude Reduced
	CMAP AMPLITUDE (mV)	3	3.3	4.8	5	3.5	3.3	1.5	1.6	
	MCV(m/s)	50.0	51.0	52.2	52.0	42.0	42 2	41.1	41.6	
	SDL (ms)	2.2	2	2.1	1.9	-		-		1. B/L Median and Ulnar Nerves SNAP Amplitude Reduced
	SNAP (µV)	52.1	51.9	16.7	16.9					
	SCV (m/S)	55.5	55.8	56.0	55.9					
	F-wave latency	1. Prolonged in B/L Median and Ulnar Nerves. 2. Absent in B/L Peroneal & Tibial Nerves.								
Case 5	DML (ms)	2.5	3.2	3.1	3.3	4.6	5.1	5.2	4.9	1. B/L Median and Ulnar Nerve DML Prolonged 2. B/L Tibial and Peroneal Nerve DML Prolonged 3. B/L Tibial and Peroneal Nerve CMAP Amplitude Reduced 4. B/L Tibial and Peroneal Nerve MVC Reduced
	CMAP AMPLITUDE (mV)	5.2	6.1	7.3	8.7	2.3	3.6	0.9	1.1	
	MCV(m/s)	56.7	58.9	52.4	54.1	32.4	28.6	26.2	31.8	
	SDL (ms)	1.2	1.6	1.3	1.1	-		-		1. B/L Median and Ulnar Nerves SDL Prolonged 2. B/L Median and Ulnar Nerves SNAP Amplitude Prolonged 3. B/L Median and Ulnar Nerves SCV Reduced 4. SNAPS are absent in B/L Sural Nerves
	SNAP (µV)	42.3	39.5	6.8	7.4					
	SCV (m/S)	43.8	46.4	23.6	31.2					
	F-wave latency	1. Prolonged in B/L Median and Ulnar Nerves. 2. Absent in B/L Peroneal & Tibial Nerves.								

Differential diagnosis

The most likely diagnosis was GBS based on the first presentation of acute flaccid polyneuropathy and an identifiable trigger in the form of recent vaccination. Spinal cord compression, peripheral neuropathy, and autoimmune disorders or vasculitis (ANCA-positive vasculitis) are a few differentials that would have presented with acute flaccid neuropathy. MRI whole spine did not show any significant finding of cord compression or annular bulges, or curvature deformity. Moreover, no bowel or bladder involvement and the absence of back pain helped rule out spinal cord compression. The classic CSF findings of albumin-cytological dissociation and NCS findings of demyelinating, sensorimotor polyneuropathy confirmed the diagnosis of GBS. In addition to these, case 1 had a significant leukocytosis, which could tilt the diagnosis towards viral meningitis, but there were no clinical evident signs for the same. Other differentials can be critical illness neuropathy [15], tick paralysis, and botulism.

## Discussion

GBS is a heterogeneous disorder characterized by rapidly progressive, symmetrical ascending paralysis with areflexia or hyporeflexia triggered by certain infections, such as *C. jejuni*, cytomegalovirus, *M. pneumoniae*, Epstein-Barr virus, and Zika virus [4-7]. Studies done in the past during the 1976 swine flu outbreak revealed that GBS developed after vaccination, with 8.8 cases per million recipients of influenza vaccine being documented [16].

A single recombinant chimpanzee adenovirus (ChAdOx1) vector with DNA encoding the S glycoprotein/spike protein of SARS-CoV-2 makes up the Oxford/AstraZeneca ChAdOx1 nCoV-19 (AZD1222) COVID-19 vaccine. The host produces a strong T-cell immunological response and provides high protection against the virus [17]. The Oxford/AstraZeneca COVID-19 vaccine is 92% effective against the Delta variant and 86% reduction in hospitalization and no death by the Alpha variant (B.1.1.7; formerly the ‘Kent’ variant) according to a Real-World Data preprint published by the Public Health England (PHE) [18].

GBS can also present with cranial nerve deficits. Pathogenesis is unclear, but molecular mimicry, complement activation, and anti-ganglioside antibody production have been implicated [19]. Back pain can also be one of the presenting complaints in GBS before the onset of weakness and can be misleading in the initial stages of diagnosis [19]. All five above-mentioned cases were diagnosed with GBS based on Brighton criteria [20]. It has seven diagnostic criteria and four levels of diagnostic certainty developed by Brighton Collaboration [21]. Amongst them, four responded well to IVIG and steroids, and one improved partially with residual neuro deficit. The latency period between the onset of symptoms of GBS and vaccination was fairly long in two patients (one month) and short (12 days) in one patient. All five patients received a non-replicating viral vector (AstraZeneca) vaccine. Limitations existed in a study conducted by Narasimhalu et al. as neither NCSs nor CSF studies had been carried out [22]. In the study of Allen et al., all four patients did not undergo a Nerve conduction study, and the outcome of those cases was not described in detail [23].

A causal relationship between GBS and COVID-19 vaccination remains speculative. Theory suggests vaccination stimulates immune response attributing to the production of antibodies and T-cells that cross-react with gangliosides at nerve membranes due to molecular mimicry [24]. Molecular mimicry requires a humoral response that requires 10-14 days to develop [22].

Josef Finister et al., in their narrative review, mentioned that though a temporal relationship between SARS-COV-2 vaccination and GBS remains unclear, more arguments are in favor than against it [8]. The committee suggests that more thorough investigations employing reliable study designs, alternate data sources, and comparisons of populations with and without vaccinations are necessary to establish the cause [25].

## Conclusions

GBS may develop time linked to the first dose of SARS COV-2 Oxford/AstraZeneca vaccine. It is still not possible to conclude a significant association between GBS and COVID-19 vaccination. Contaminated proteins may elicit anti-ganglioside antibodies; hence increasing purification and filtration steps can reduce the risk of vaccine-associated GBS. Although there is no evidence that the COVID-19 vaccine increases the risk of GBS, it is nevertheless important to keep a high level of suspicion, and potential side effects related to the vaccine should be reported.
